# 4-Nitroaniline–2,4,6-trimethoxybenz­aldehyde (1/1)

**DOI:** 10.1107/S1600536810023664

**Published:** 2010-06-23

**Authors:** Abdullah M. Asiri, Salman A. Khan, Kong Wai Tan, Seik Weng Ng

**Affiliations:** a Chemistry Department, Faculty of Science, King Abdul Aziz University, PO Box 80203, Jeddah 21589, Saudi Arabia; b Department of Chemistry, University of Malaya, 50603 Kuala Lumpur, Malaysia

## Abstract

In the title co-crystal, C_6_H_6_N_2_O_2._C_10_H_12_O_4_, the two components are held together by an N—H⋯O_aldehyde_ hydrogen bond. Adjacent co-crystals are linked by weaker N—H⋯O_nitro_ hydrogen bonds, forming a linear chain. The two aromatic rings of the components are aligned at 75.2 (1)°. The crystal studied was a non-merohedral twin with a 24% minor component.

## Related literature

For some examples of co-crystals of 4-nitro­aniline, see: Bertolasi *et al.* (2001 ▶); Dederer & Gieren (1979 ▶); Huang *et al.* (1996 ▶); Koshima *et al.* (1996 ▶); Rashid & Deschamps (2006 ▶); Singh *et al.* (2003 ▶); Smith *et al.* (1997 ▶); Weber (1981 ▶); Zaitu *et al.* (1995 ▶). For the treatment of non-merohedral twins, see: Spek (2009 ▶).
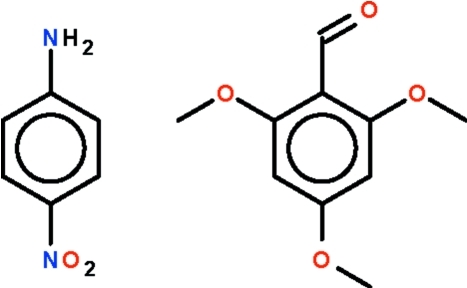


## Experimental

### 

#### Crystal data

C_6_H_6_N_2_O_2_·C_10_H_12_O_4_
*M* *_r_* = 334.32
Monoclinic, 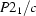

*a* = 7.4409 (11) Å
*b* = 30.022 (5) Å
*c* = 6.9400 (11) Å
β = 93.237 (3)°
*V* = 1547.9 (4) Å^3^
*Z* = 4
Mo *K*α radiation
μ = 0.11 mm^−1^
*T* = 100 K
0.15 × 0.10 × 0.05 mm

#### Data collection

Bruker SMART APEX diffractometer
8127 measured reflections
2722 independent reflections
1834 reflections with *I* > 2σ(*I*)
*R*_int_ = 0.059

#### Refinement

*R*[*F* ^2^ > 2σ(*F* ^2^)] = 0.049
*wR*(*F* ^2^) = 0.124
*S* = 1.01
2722 reflections
221 parameters
H-atom parameters constrained
Δρ_max_ = 0.22 e Å^−3^
Δρ_min_ = −0.41 e Å^−3^

### 

Data collection: *APEX2* (Bruker, 2009 ▶); cell refinement: *SAINT* (Bruker, 2009 ▶); data reduction: *SAINT*; program(s) used to solve structure: *SHELXS97* (Sheldrick, 2008 ▶); program(s) used to refine structure: *SHELXL97* (Sheldrick, 2008 ▶); molecular graphics: *X-SEED* (Barbour, 2001 ▶); software used to prepare material for publication: *publCIF* (Westrip, 2010 ▶).

## Supplementary Material

Crystal structure: contains datablocks global, I. DOI: 10.1107/S1600536810023664/si2271sup1.cif

Structure factors: contains datablocks I. DOI: 10.1107/S1600536810023664/si2271Isup2.hkl

Additional supplementary materials:  crystallographic information; 3D view; checkCIF report

## Figures and Tables

**Table 1:** Hydrogen-bond geometry (Å, °)

*D*—H⋯*A*	*D*—H	H⋯*A*	*D*⋯*A*	*D*—H⋯*A*
N1—H12⋯O1	0.86	2.16	3.016 (3)	172
N1—H11⋯O5^i^	0.86	2.50	3.288 (3)	152
N1—H11⋯O6^i^	0.86	2.50	3.293 (3)	154

Symmetry code: (i) 
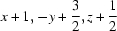
.

**Fig. 1.**
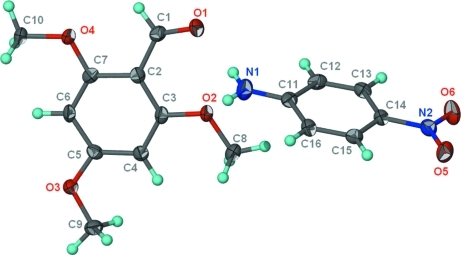
Thermal ellipsoid plot (Barbour, 2001) of the C6H6N2O2–C10H12O4 co-crystal at the 70% probability level; hydrogen atoms are drawn as spheres of arbitrary radius.

## supplementary crystallographic information

### Comment

Aromatic aldehydes readily condense with aromatic amines to yield Schiff bases. However, 2,4,6-trimethoxylbenzaldehyde and 4-nitroaniline reactants did not condense but instead co-crystallized in the attempted synthesis. The condensation probably did not proceed owing to the decreased basicity of the amino group, which is situated opposite the electron-withdrawing nitro group in the aromatic ring. The co-crystal (Scheme I, Fig. 1) has the components linked by an H–N···O hydrogen bond; the two aromatic rings aligned at 75.2 (1) °. Adjacent co-crystals are linked by weaker *N*–*H*···O_nitro_ hydrogen bonds to form a linear chain.

4-Nitroaniline forms a number of co-crystals with other neutral compounds; for their description, see: Bertolasi *et al.* (2001); Dederer & Gieren (1979); Huang *et al.* (1996); Koshima *et al.* (1996); Rashid & Deschamps (2006); Singh *et al.* (2003); Smith *et al.* (1997); Weber (1981); Zaitu *et al.* (1995).

### Experimental

2,4, 6-Trimethoxybenzaldehyde (0.50 g, 3.3 mmol) and 4-nitroaniline (0.57 g, 3.3 mmol) were heated in methanol (15 ml) for 5 h. Yellow crystals separated from the cool solution after a day.

### Refinement

Carbon- and nitrogen-bound H-atoms were placed in calculated positions [C–H 0.95 to 0.98 Å, N–H 0.86 Å; *U*(H) 1.2 to 1.5*U*_eq_(*C*,*N*)] and were included in the refinement in the riding model approximation.

The crystal studied is a non-merohedral twin; the twin law (1 0 0.121, 0 - 1 0, 0 0 - 1) as given by *PLATON *(Spek, 2009) was used to de-twin the diffraction data.

### Crystal data

**Table:**

C_6_H_6_N_2_O_2_·C_10_H_12_O_4_	*F*(000) = 704
*M**_r_* = 334.32	*D*_x_ = 1.435 Mg m^−^^3^
Monoclinic, *P*2_1_/*c*	Mo *K*α radiation, λ = 0.71073 Å
Hall symbol: -P 2ybc	Cell parameters from 1019 reflections
*a* = 7.4409 (11) Å	θ = 2.7–24.4°
*b* = 30.022 (5) Å	µ = 0.11 mm^−^^1^
*c* = 6.9400 (11) Å	*T* = 100 K
β = 93.237 (3)°	Prism, yellow
*V* = 1547.9 (4) Å^3^	0.15 × 0.10 × 0.05 mm
*Z* = 4	

### Data collection

**Table:**

Bruker SMART APEX diffractometer	1834 reflections with *I* > 2σ(*I*)
Radiation source: fine-focus sealed tube	*R*_int_ = 0.059
graphite	θ_max_ = 25.0°, θ_min_ = 1.4°
ω scans	*h* = −8→8
8127 measured reflections	*k* = −32→35
2722 independent reflections	*l* = −8→8

### Refinement

**Table:**

Refinement on *F*^2^	Primary atom site location: structure-invariant direct methods
Least-squares matrix: full	Secondary atom site location: difference Fourier map
*R*[*F*^2^ > 2σ(*F*^2^)] = 0.049	Hydrogen site location: inferred from neighbouring sites
*wR*(*F*^2^) = 0.124	H-atom parameters constrained
*S* = 1.01	*w* = 1/[σ^2^(*F*_o_^2^) + (0.0537*P*)^2^ + 0.141*P*] where *P* = (*F*_o_^2^ + 2*F*_c_^2^)/3
2722 reflections	(Δ/σ)_max_ = 0.001
221 parameters	Δρ_max_ = 0.22 e Å^−^^3^
0 restraints	Δρ_min_ = −0.41 e Å^−^^3^

### Fractional atomic coordinates and isotropic or equivalent isotropic displacement parameters (Å^2^)

**Table:**

	*x*	*y*	*z*	*U*_iso_*/*U*_eq_	
O1	0.7019 (2)	0.57392 (6)	0.7861 (3)	0.0238 (5)	
O2	0.7670 (2)	0.61120 (6)	0.4418 (2)	0.0182 (4)	
O3	1.3371 (2)	0.57367 (6)	0.1850 (2)	0.0203 (4)	
O4	1.1646 (2)	0.51253 (6)	0.7725 (2)	0.0176 (4)	
O5	0.0363 (2)	0.80574 (6)	0.5273 (3)	0.0266 (5)	
O6	−0.0908 (2)	0.74324 (7)	0.4450 (3)	0.0291 (5)	
N1	0.6593 (3)	0.67379 (7)	0.7727 (3)	0.0233 (5)	
H11	0.7505	0.6884	0.8209	0.028*	
H12	0.6631	0.6452	0.7670	0.028*	
N2	0.0398 (3)	0.76456 (7)	0.5159 (3)	0.0193 (5)	
C1	0.8433 (3)	0.55435 (8)	0.7589 (4)	0.0177 (6)	
H1	0.8764	0.5314	0.8482	0.021*	
C2	0.9658 (3)	0.56212 (8)	0.6066 (3)	0.0152 (6)	
C3	0.9285 (3)	0.59010 (8)	0.4462 (4)	0.0154 (6)	
C4	1.0482 (3)	0.59515 (8)	0.3011 (4)	0.0155 (6)	
H4	1.0203	0.6140	0.1936	0.019*	
C5	1.2098 (3)	0.57189 (8)	0.3175 (4)	0.0157 (6)	
C6	1.2542 (3)	0.54410 (8)	0.4744 (4)	0.0170 (6)	
H6	1.3662	0.5289	0.4834	0.020*	
C7	1.1335 (3)	0.53914 (8)	0.6155 (3)	0.0144 (6)	
C8	0.7184 (3)	0.63908 (9)	0.2781 (4)	0.0216 (6)	
H8A	0.5983	0.6516	0.2923	0.032*	
H8B	0.7179	0.6213	0.1597	0.032*	
H8C	0.8061	0.6633	0.2709	0.032*	
C9	1.3034 (3)	0.60080 (8)	0.0177 (4)	0.0198 (6)	
H9A	1.4039	0.5980	−0.0669	0.030*	
H9B	1.2913	0.6320	0.0570	0.030*	
H9C	1.1920	0.5910	−0.0516	0.030*	
C10	1.3340 (3)	0.48903 (9)	0.7879 (4)	0.0193 (6)	
H10A	1.3403	0.4709	0.9054	0.029*	
H10B	1.4332	0.5106	0.7934	0.029*	
H10C	1.3436	0.4697	0.6751	0.029*	
C11	0.5099 (3)	0.69579 (9)	0.7057 (3)	0.0168 (6)	
C12	0.3577 (3)	0.67245 (9)	0.6292 (4)	0.0189 (6)	
H12A	0.3622	0.6409	0.6183	0.023*	
C13	0.2034 (3)	0.69466 (9)	0.5705 (4)	0.0186 (6)	
H13	0.1006	0.6786	0.5216	0.022*	
C14	0.1986 (3)	0.74094 (8)	0.5832 (4)	0.0165 (6)	
C15	0.3483 (3)	0.76498 (8)	0.6542 (3)	0.0162 (6)	
H15	0.3440	0.7966	0.6610	0.019*	
C16	0.5015 (3)	0.74254 (9)	0.7139 (4)	0.0176 (6)	
H16	0.6040	0.7588	0.7617	0.021*	

### Atomic displacement parameters (Å^2^)

**Table:**

	*U*^11^	*U*^22^	*U*^33^	*U*^12^	*U*^13^	*U*^23^
O1	0.0193 (10)	0.0228 (11)	0.0302 (11)	0.0076 (8)	0.0082 (8)	0.0052 (9)
O2	0.0152 (9)	0.0201 (10)	0.0195 (10)	0.0065 (8)	0.0028 (7)	0.0045 (8)
O3	0.0188 (10)	0.0228 (11)	0.0200 (10)	0.0060 (8)	0.0070 (8)	0.0085 (8)
O4	0.0163 (9)	0.0172 (10)	0.0195 (10)	0.0054 (7)	0.0023 (7)	0.0049 (8)
O5	0.0257 (11)	0.0204 (12)	0.0337 (12)	0.0076 (9)	0.0015 (8)	0.0010 (9)
O6	0.0173 (10)	0.0360 (13)	0.0332 (12)	−0.0005 (9)	−0.0068 (9)	−0.0050 (10)
N1	0.0213 (12)	0.0170 (13)	0.0311 (13)	0.0035 (10)	−0.0030 (10)	−0.0009 (11)
N2	0.0183 (12)	0.0219 (14)	0.0178 (12)	0.0011 (10)	0.0028 (9)	−0.0001 (10)
C1	0.0207 (14)	0.0123 (14)	0.0201 (14)	0.0010 (12)	0.0005 (11)	0.0014 (11)
C2	0.0160 (13)	0.0131 (14)	0.0165 (13)	−0.0013 (10)	0.0012 (10)	−0.0016 (11)
C3	0.0133 (13)	0.0136 (14)	0.0189 (13)	0.0016 (11)	−0.0017 (10)	−0.0027 (11)
C4	0.0157 (13)	0.0139 (14)	0.0171 (13)	−0.0008 (10)	0.0015 (10)	−0.0010 (11)
C5	0.0154 (13)	0.0147 (14)	0.0174 (13)	−0.0017 (11)	0.0039 (10)	−0.0015 (11)
C6	0.0136 (13)	0.0142 (14)	0.0230 (14)	0.0022 (11)	−0.0003 (11)	0.0002 (11)
C7	0.0190 (14)	0.0098 (14)	0.0141 (13)	−0.0002 (10)	−0.0012 (10)	−0.0018 (11)
C8	0.0207 (14)	0.0211 (16)	0.0227 (14)	0.0046 (12)	−0.0017 (11)	0.0062 (12)
C9	0.0239 (15)	0.0173 (15)	0.0185 (14)	0.0015 (11)	0.0035 (11)	0.0044 (12)
C10	0.0148 (13)	0.0199 (15)	0.0230 (14)	0.0057 (11)	0.0004 (11)	0.0045 (12)
C11	0.0205 (14)	0.0186 (15)	0.0118 (13)	0.0037 (11)	0.0052 (11)	−0.0005 (11)
C12	0.0246 (14)	0.0121 (14)	0.0200 (14)	−0.0020 (12)	0.0025 (11)	0.0003 (11)
C13	0.0175 (14)	0.0218 (16)	0.0166 (14)	−0.0044 (12)	0.0029 (11)	−0.0013 (12)
C14	0.0154 (13)	0.0175 (15)	0.0168 (13)	0.0009 (11)	0.0011 (10)	0.0009 (11)
C15	0.0212 (14)	0.0122 (14)	0.0158 (13)	−0.0003 (11)	0.0056 (11)	−0.0014 (11)
C16	0.0165 (13)	0.0210 (16)	0.0152 (13)	0.0000 (11)	0.0011 (11)	−0.0023 (11)

### Geometric parameters (Å, °)

**Table:**

O1—C1	1.229 (3)	C6—C7	1.374 (3)
O2—C3	1.357 (3)	C6—H6	0.9500
O2—C8	1.441 (3)	C8—H8A	0.9800
O3—C5	1.358 (3)	C8—H8B	0.9800
O3—C9	1.428 (3)	C8—H8C	0.9800
O4—C7	1.360 (3)	C9—H9A	0.9800
O4—C10	1.444 (3)	C9—H9B	0.9800
O5—N2	1.239 (3)	C9—H9C	0.9800
O6—N2	1.242 (3)	C10—H10A	0.9800
N1—C11	1.352 (3)	C10—H10B	0.9800
N1—H11	0.8600	C10—H10C	0.9800
N1—H12	0.8600	C11—C16	1.406 (4)
N2—C14	1.434 (3)	C11—C12	1.410 (3)
C1—C2	1.454 (3)	C12—C13	1.370 (3)
C1—H1	0.9500	C12—H12A	0.9500
C2—C3	1.409 (3)	C13—C14	1.393 (3)
C2—C7	1.424 (3)	C13—H13	0.9500
C3—C4	1.390 (3)	C14—C15	1.394 (3)
C4—C5	1.389 (3)	C15—C16	1.368 (3)
C4—H4	0.9500	C15—H15	0.9500
C5—C6	1.397 (3)	C16—H16	0.9500
			
C3—O2—C8	118.10 (19)	O2—C8—H8C	109.5
C5—O3—C9	118.39 (19)	H8A—C8—H8C	109.5
C7—O4—C10	117.03 (19)	H8B—C8—H8C	109.5
C11—N1—H11	120.0	O3—C9—H9A	109.5
C11—N1—H12	120.0	O3—C9—H9B	109.5
H11—N1—H12	120.0	H9A—C9—H9B	109.5
O5—N2—O6	121.4 (2)	O3—C9—H9C	109.5
O5—N2—C14	119.5 (2)	H9A—C9—H9C	109.5
O6—N2—C14	119.1 (2)	H9B—C9—H9C	109.5
O1—C1—C2	127.8 (2)	O4—C10—H10A	109.5
O1—C1—H1	116.1	O4—C10—H10B	109.5
C2—C1—H1	116.1	H10A—C10—H10B	109.5
C3—C2—C7	117.2 (2)	O4—C10—H10C	109.5
C3—C2—C1	124.5 (2)	H10A—C10—H10C	109.5
C7—C2—C1	118.3 (2)	H10B—C10—H10C	109.5
O2—C3—C4	122.4 (2)	N1—C11—C16	120.7 (2)
O2—C3—C2	115.5 (2)	N1—C11—C12	120.9 (2)
C4—C3—C2	122.1 (2)	C16—C11—C12	118.3 (2)
C3—C4—C5	118.2 (2)	C13—C12—C11	120.8 (2)
C3—C4—H4	120.9	C13—C12—H12A	119.6
C5—C4—H4	120.9	C11—C12—H12A	119.6
O3—C5—C4	123.9 (2)	C12—C13—C14	119.4 (2)
O3—C5—C6	114.1 (2)	C12—C13—H13	120.3
C4—C5—C6	122.0 (2)	C14—C13—H13	120.3
C7—C6—C5	119.0 (2)	C15—C14—C13	121.1 (2)
C7—C6—H6	120.5	C15—C14—N2	119.1 (2)
C5—C6—H6	120.5	C13—C14—N2	119.7 (2)
O4—C7—C6	123.1 (2)	C16—C15—C14	119.2 (2)
O4—C7—C2	115.4 (2)	C16—C15—H15	120.4
C6—C7—C2	121.5 (2)	C14—C15—H15	120.4
O2—C8—H8A	109.5	C15—C16—C11	121.2 (2)
O2—C8—H8B	109.5	C15—C16—H16	119.4
H8A—C8—H8B	109.5	C11—C16—H16	119.4
			
O1—C1—C2—C3	−9.3 (4)	C5—C6—C7—C2	−0.9 (4)
O1—C1—C2—C7	172.5 (2)	C3—C2—C7—O4	179.9 (2)
C8—O2—C3—C4	1.2 (3)	C1—C2—C7—O4	−1.8 (3)
C8—O2—C3—C2	−177.9 (2)	C3—C2—C7—C6	0.2 (3)
C7—C2—C3—O2	179.5 (2)	C1—C2—C7—C6	178.6 (2)
C1—C2—C3—O2	1.3 (4)	N1—C11—C12—C13	176.7 (2)
C7—C2—C3—C4	0.5 (3)	C16—C11—C12—C13	−2.2 (4)
C1—C2—C3—C4	−177.7 (2)	C11—C12—C13—C14	1.3 (4)
O2—C3—C4—C5	−179.5 (2)	C12—C13—C14—C15	0.2 (4)
C2—C3—C4—C5	−0.5 (4)	C12—C13—C14—N2	177.8 (2)
C9—O3—C5—C4	−0.4 (3)	O5—N2—C14—C15	−2.2 (3)
C9—O3—C5—C6	179.3 (2)	O6—N2—C14—C15	176.9 (2)
C3—C4—C5—O3	179.4 (2)	O5—N2—C14—C13	−179.9 (2)
C3—C4—C5—C6	−0.2 (4)	O6—N2—C14—C13	−0.8 (3)
O3—C5—C6—C7	−178.8 (2)	C13—C14—C15—C16	−0.6 (4)
C4—C5—C6—C7	0.9 (4)	N2—C14—C15—C16	−178.3 (2)
C10—O4—C7—C6	0.3 (3)	C14—C15—C16—C11	−0.3 (4)
C10—O4—C7—C2	−179.4 (2)	N1—C11—C16—C15	−177.2 (2)
C5—C6—C7—O4	179.5 (2)	C12—C11—C16—C15	1.7 (4)

### Hydrogen-bond geometry (Å, °)

**Table:**

*D*—H···*A*	*D*—H	H···*A*	*D*···*A*	*D*—H···*A*
N1—H12···O1	0.86	2.16	3.016 (3)	172
N1—H11···O5^i^	0.86	2.50	3.288 (3)	152
N1—H11···O6^i^	0.86	2.50	3.293 (3)	154

Symmetry codes: (i) *x*+1, −*y*+3/2, *z*+1/2.
